# Gastric cancer with positive peritoneal cytology: survival benefit after induction chemotherapy and conversion to negative peritoneal cytology

**DOI:** 10.1186/s12957-021-02351-x

**Published:** 2021-08-17

**Authors:** Massimiliano Valletti, Dilmurodjon Eshmuminov, Nicola Gnecco, Christian Alexander Gutschow, Paul Magnus Schneider, Kuno Lehmann

**Affiliations:** 1 Department of Surgery and Transplantation, University Hospital Zurich, Zurich, Switzerland; 2 Research Center for Statistics, University of Geneva, Geneva, Switzerland

**Keywords:** Gastric cancer, Peritoneal lavage cytology, Peritoneal metastasis, Neoadjuvant chemotherapy

## Abstract

**Background:**

The optimal treatment in patients with gastric cancer and peritoneal disease remains controversial. Some guidelines indicate palliative treatment only, while others consider surgical treatment in case of positive lavage cytology (CY+) or limited peritoneal disease. Here, we analyzed the role of peritoneal disease in patients with gastric cancer, and the prognostic relevance of response to neoadjuvant therapy.

**Methods:**

In this retrospective cohort analysis, we analyzed patients with adenocarcinoma of the stomach or esophago-gastric junction from a single center operated between 2011 and 2019. According to histology and lavage cytology, patients were classified into four risk groups: (A) no peritoneal disease, (B) CY+ who converted to negative lavage cytology (CY−) after neoadjuvant chemotherapy, (C) CY+ without conversion after chemotherapy, and (D) patients with visible peritoneal metastasis.

**Results:**

Overall, *n* = 172 patients were included. At initial presentation, *n* = 125 (73%) had no peritoneal disease, and about a third of patients (*n* = 47, 27%) had microscopic or macroscopic peritoneal disease. Among them, *n* = 14 (8%) were CY+ without visible peritoneal metastasis, *n* = 9 converted to CY− after chemotherapy, and in *n* = 5 no conversion was observed. Median overall survival was not reached in patients who had initially no peritoneal disease and in patients who converted after chemotherapy, resulting in 3-year survival rates of 65% and 53%. In contrast, median overall survival was reduced to 13 months (95% CI 8.7–16.7) in patients without conversion and was 16 months (95% CI 12–20.5) in patients with peritoneal metastasis without difference between the two groups (*p* = .364). The conversion rate from CY+ to CY− was significantly higher after neoadjuvant treatment with FLOT (5-fluorouracil plus leucovorin, oxaliplatin, and docetaxel) compared to ECF (epirubicin, cisplatin, and 5-fluorouracil) (*p* = 0.027).

**Conclusion:**

Conversion of CY+ to CY− after neoadjuvant chemotherapy with FLOT is a significant prognostic factor for a better overall survival. Surgical treatment in well-selected patients should therefore be considered. However, peritoneal recurrence remains frequent despite conversion, urging for a better local control.

## Introduction

Gastric cancer is a leading cause of cancer death worldwide, with an estimated 5-year survival rate of 26% in Europe [1]. According to the GLOBOCAN 2018 estimates of cancer incidence, gastric cancer is the 5th most common neoplasm and the 3rd most deadly cancer worldwide, with 783,000 deaths in 2018 [2]. Due to the lack of national screening programs in Western countries, many patients are diagnosed with advanced stage disease and up to 20% present with peritoneal metastasis upon open or laparoscopic exploration [3, 4]. Peritoneal metastases have a dismal prognosis, and most patients receive palliative chemotherapy only [5]. In contrast, the relevance of microscopic disease, detectable only as CY+ remains unclear [6]. There is an ongoing debate if these patients should undergo gastrectomy [7, 8], and the available national guidelines are not consistent regarding treatment recommendations. For example, the National Comprehensive Cancer Network (NCCN) gastric cancer guidelines define CY+ as metastatic (M1) disease and recommend a palliative treatment [9], similar to the Japanese Gastric Cancer Association, which classifies positive peritoneal cytology as M1 and does not recommend surgery [10, 11]. In contrast, European guidelines, e.g., the German S3, or the ESMO guidelines are less detailed and remain unclear, probably due to the lack of data in Western patients [12, 13]. Another open question is how to deal with patients who had CY+ and converted to CY− after neoadjuvant treatment. Reports from Japan show an improved survival of this subgroup and therefore recommend a more radical treatment after conversion to CY− [14, 15]. The literature is scarce for Western patients [16], while novel treatment options for patients with locally advanced disease are available, e.g., the radical cytoreductive surgery (CRS) with hyperthermic intraperitoneal chemotherapy (HIPEC) [17] or the pressurized intraperitoneal aerosol chemotherapy (PIPAC) [18]. In this study, we analyzed the overall survival of gastric cancer with a focus on patients with CY+.

## Materials and methods

### Patients

Patients with gastric cancer treated at the University Hospital of Zurich between September 2011 and December 2019 were retrospectively analyzed. All patients were discussed in an interdisciplinary tumor board. Neoadjuvant systemic treatment was performed according to international standards. Patients received FLOT (5-fluorouracil plus leucovorin, oxaliplatin and docetaxel) or ECF (Epirubicin, cisplatin and 5-fluorouracil) depending on the preference of the treating medical oncologist. Surgery was performed with either total or subtotal gastrectomy and D2-lymphadenectomy 3 to 4 weeks after completion of neoadjuvant systemic treatment. Follow-up included clinical exams, upper endoscopy, and CT scan.

### Peritoneal lavage cytology

Laparoscopy with cytology was performed to evaluate for peritoneal disease when considering chemotherapy or surgery. Suspicious macroscopic peritoneal implants detected during laparoscopy were biopsied. If peritoneal seeding was confirmed histologically, palliative treatment was considered. The second peritoneal lavage for cytology was performed after neoadjuvant systemic treatment or at the time of surgical resection. One thousand milliliters of saline was installed into the upper and lower abdomen without manipulation of the tumor. After a 10-min incubation period, all available fluid was aspirated and sent for cytological analysis. Cytological evaluation included microscopy and immunohistochemistry.

### Data analysis

Statistical analysis was performed using R statistical software (version 4.0.2.). *p*-values are based on the chi-square test and Fischer’s exact test for categorical variables, and ANOVA for continuous variables, and were considered statistically significant if < 0.05. The Kaplan-Meier method was used to calculate survival. Groups were compared using the log-rank test. Follow-up time was defined from the date of diagnosis to the following event: 1) death or last follow-up day and 2) documented evidence of cancer recurrence by histology or imaging. Patients with CY− and clinical absence of peritoneal metastasis (negative group) were used as the control group.

## Results

### Patient characteristics

One hundred and seventy-two patients were analyzed with a mean follow-up of 25 (± 18.1) months. According to histology and lavage cytology, patients were classified into four risk groups: (A) no peritoneal disease (negative group *n* = 125), (B) CY+ who converted to CY− after neoadjuvant chemotherapy (converted group *n* = 9), (C) CY+ without conversion to CY− after neoadjuvant chemotherapy (non-converted group *n* = 5), and (D) patients with visible peritoneal metastasis (*n* = 33). Patient characteristics are shown in Table 1.

**Table 1:** Patient characteristics

	Negative***, n*** = 125	Converted negative, ***n*** = 9	Not converted, ***n*** = 5	Peritoneal metastasis, ***n*** = 33	***p***
Age (years)	61 ± 13	54 ± 9	64 ± 9	53 ± 15	0.016
Gender m/f	88 (70%)/7 (30%)	4 (44%)/5 (56%)	3 (60%)/2 (40%)	21 (64%)/2 (36%)	0.390
**Tumor location**					
AEG	61 (49%)	5 (55%)	5 (100%)	10 (30%)	0.021
Gastric	64 (51%)	4 (45%)	0	23 (70%)	
**Chemotherapy**					
FLOT	62 (50%)	9 (100%)	2 (40%)	19 (58%)	>0.001
ECF	13 (10%)	0	3 (60%)	4 (12%)	
Others	0	0	0	3 (9%)	
No chemotherapy	50 (40%)	0	0	7 (21%)	
**pT or ypT**					
T0	7 (5%)	1 (11%)	0	NA	0.141
T1	36 (29%)	0	0		
T2	20 (16%)	1 (11%)	0		
T3	51 (41%)	7 (78%)	4 (80%)		
T4	11 (9%)	0	1 (20%)		
**Positive lymph nodes**	64 (51%)	5 (55%)	5 (100%)	NA	0.109
Mean retrieved LN	42 ± 20	56 ± 11	36 ± 11	NA	0.105
**Grading**^a^					
G1	3 (3%)	0	0	0	0.772
G2	25 (26%)	1 (11%)	1 (20%)	2 (13%)	
G3	67 (71%)	8 (89%)	4 (80%)	13 (87%)	
**Lauren type**^a^					
Intestinal	64 (61%)	4 (45%)	4 (80%)	6 (22%)	0.002
Diffuse	41 (39%)	5 (55%)	1 (20%)	21 (78%)	

Patient characteristics are summarized according to the presence or absence of peritoneal disease during staging laparoscopy. While *n* = 125 patients had no peritoneal disease, *n* = 9 converted to CY− and *n* = 5 remained CY+ after neoadjuvant chemotherapy. Macroscopic peritoneal metastasis was present in *n* = 33 patients

*AEG* adenocarcinoma of the esophago-gastric junction, *LN* lymph nodes, *NA* not available

^a^The data for some patients is missing. *p-*values are based on the chi-square test and Fisher’s exact test for categorical variables and ANOVA for continuous variables

### Response to neoadjuvant chemotherapy

All patients with no peritoneal disease and conversion to CY− and those who remained CY+ underwent radical surgery. Patients with positive peritoneal cytology received FLOT in 79% (11/14) and ECF in 21% (3/14) of patients. We observed a higher conversion rate to CY− after treatment with FLOT compared to ECF (9/11 vs. 0/3; *p* = .027). Progression during chemotherapy was observed in five patients (*n* = 3 patients with ECF, *n* = 2 with FLOT), resulting in a CY+ after neoadjuvant systemic treatment. Among 33 patients with peritoneal metastasis, 28 received palliative chemotherapy and five patients underwent palliative surgery.

### Overall survival

**Fig. 1:**
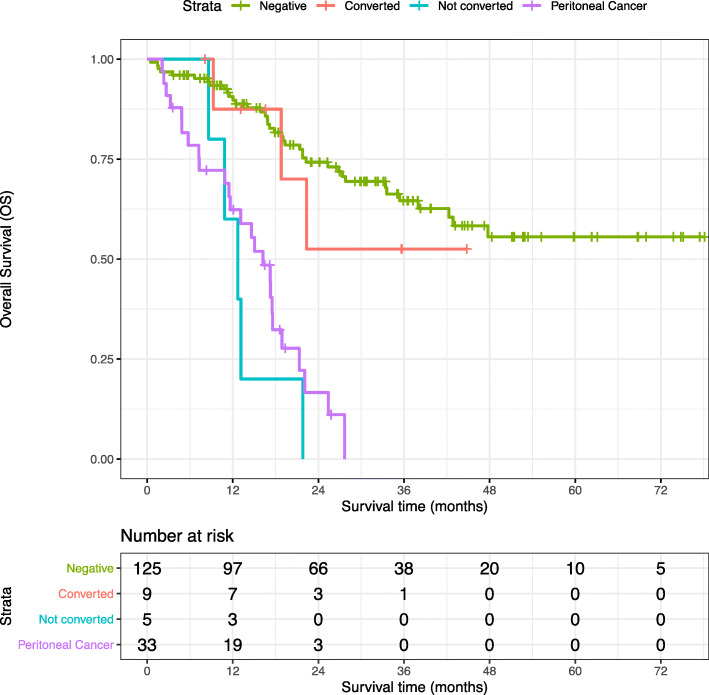
Overall survival. Kaplan-Meier plot for patients according to their peritoneal disease status. Median OS of patients without peritoneal disease and of patients who did convert after neoadjuvant therapy was not reached. In contrast, median OS was reduced to 13 months (95% CI 8.7–16.7) in patients without conversion and was 16 months (95% CI 12–20.5) in patients with peritoneal metastasis without difference between the two groups (*p* = .364)

Conversion to CY− after neoadjuvant chemotherapy resulted in an improved overall survival (Fig. 1). Median overall survival was not reached in patients without peritoneal disease and in patients who converted to CY− after chemotherapy, resulting in 3-year survival rates of 65% and 53%. Median overall survival was reduced to 13 months (95% CI 8.7–16.7) in patients without conversion and was 16 months (95% CI 12–20.5) in patients with peritoneal metastasis without difference between the two groups (*p* = 0.36). Overall survival of patients after conversion to CY− was significantly better compared to patients who did not convert to CY− after chemotherapy (*p* = 0.01).

### Disease-free survival

**Fig. 2:**
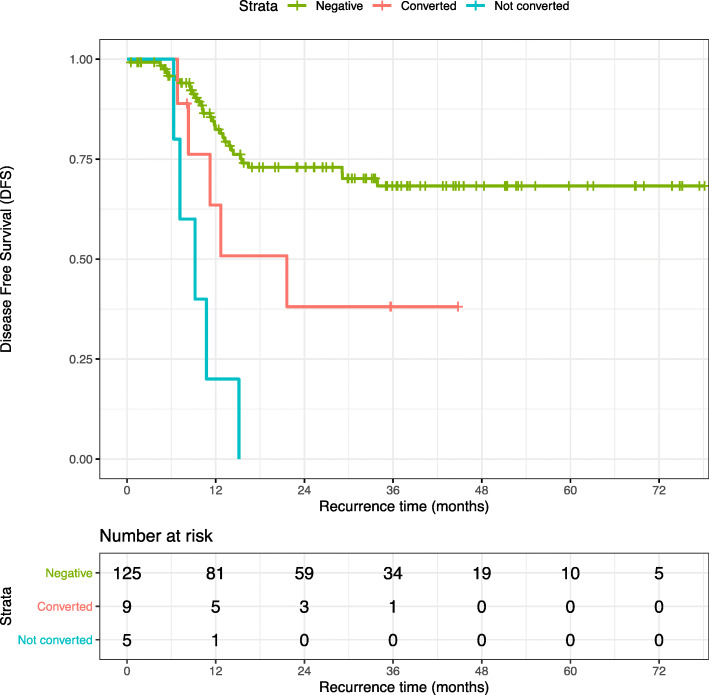
Disease-free survival. Kaplan-Meier plot for patients according to their peritoneal disease status. Median DFS at 1 year was 82% for patients without peritoneal disease and 64% for patients after conversion, with a median DFS not reached in the negative group and 21.7 months (95% CI 7.9–35) in the converted group. In contrast, DFS at 1 year was only 20% in patients with persistent positive lavage cytology after systemic therapy

Median disease-free survival was not reached in patients without peritoneal disease and was 21.7 months after conversion, resulting in 82% and 64% of patients remaining free from disease at 1-year follow-up (Fig. 2). In contrast, median disease-free survival at 1 year was only 20% in patients with persistent positive lavage cytology, highlighting a rapid disease progression beyond positive lavage cytology in the majority of the patients. Over the follow-up time of 24 months, peritoneal recurrence was finally observed in 25% (31/125) of patients who had no peritoneal disease upfront and in 55% (5/9) of patients who converted to CY− after neoadjuvant treatment.

## Discussion

Peritoneal metastasis is one of the most common forms of metastasis in patients with gastric cancer reported in the literature between 10 and 20% [3, 4, 19]. Therefore, an accurate diagnosis remains crucial for prognostic evaluations and therapeutic decisions. Staging laparoscopy and peritoneal cytology are recommended tools that allow to detect occult peritoneal spreading, especially in patients in an early stage of the disease, presenting in almost 11% of the cases with a positive peritoneal cytology [20, 21]. In this study, we could confirm the prognostic impact of peritoneal disease and we could demonstrate that patients who converted to CY− after systemic treatment have a better cumulative survival compared with patients demonstrating persisting CY+. However, the high incidence of peritoneal recurrence despite response to neoadjuvant chemotherapy remains a problem and underlines the need for better local control and novel strategies.

The role of surgery in patients with conversion after neoadjuvant systemic treatment is controversially reported in the current literature. A systematic review revealed only three studies with neoadjuvant systemic treatment in patients with positive peritoneal cytology [6], and the time point of peritoneal cytology and inclusion criteria varied considerably among these three studies. For example, one study reported 39 patients with positive peritoneal cytology without peritoneal metastasis with a median survival of 12.8 months [22]. In this study, peritoneal cytology was performed only prior to neoadjuvant systemic treatment and not thereafter. Therefore, a difference between patients with or without conversion after neoadjuvant systemic treatment was not analyzed. Another group reported 48 patients with repeated cytology, and among them, 27 converted to negative cytology, while 21 did not [8]. They observed no benefit of surgery after conversion to negative lavage cytology. In two other studies, the benefit of surgery in patients after conversion to negative lavage cytology was clearly demonstrated [14, 16]. These two studies highlight the potential risk of developing CY+ during neoadjuvant systemic treatment in patients who were initially CY−. Unfortunately, there are no known risk factors predicting poor response to neoadjuvant treatment. Such patients would profit from an early response evaluation and discontinuation of neoadjuvant systemic treatment [23]. Some authors suggest that non-responders would profit from early surgery. However, in our study, we observed that patients without conversion to CY− after systemic treatment had a very bad prognosis, on average worse than patients with macroscopic peritoneal cancer. Despite the relatively small number of patients in our series and the absence of statistical difference between the two groups, this finding likely reflects the probably unfavorable and rapid tumor biology in this situation and should call for caution.

Regarding neoadjuvant treatment, in the past decades, many advances in perioperative chemotherapy for gastric cancer have been made. For example, two clinical trials which represented landmarks regarding the topic were the MAGIC trial and the French FNCLCC/FFCD 9703 study [24, 25]. Despite a great effort, the outcome for gastric cancer remained insufficient. Furthermore, several studies reported improved survival rates with the addition of taxane-based regimens in patients with advanced gastric cancer as well as with peritoneal metastasis. For example, Wang et al. described an overall response rate of 50% for taxane-based combination chemotherapy in patients with advanced gastric cancer [26]. Moreover, Fujiwara et al. described a highly effective and well-tolerated combination of intraperitoneal and systemic chemotherapy based on docetaxel, showing that 59% of the patients displayed major response and 56% showed negative results on peritoneal cytology and no macroscopic peritoneal metastasis after treatment [27]. Another interesting work by Fushida et al. shows an overall response rate of 22% and a conversion to CY− in 81% of the patients after administration of intraperitoneal docetaxel plus S-1 for gastric cancer with peritoneal carcinomatosis [28]. In one randomized phase II trial, docetaxel in combination with 5-fluorouracil demonstrated a better overall response rate compared to ECF in patients with advanced cancer (37.8% vs. 35%) [29]. In another phase II randomized trial, the addition of cisplatin to docetaxel and 5-fluorouracil showed a favoring effect with improved response rates of docetaxel in combination with cisplatin and 5-fluorouracil over ECF or docetaxel with 5-fluorouracil, although the study was not powered sufficiently to show superiority (36.6% vs. 25% vs. 18.5%) [30]. Recently, Al-Batran and his group set a new cornerstone with the FLOT4 trial, demonstrating the superiority of FLOT over ECF/X as perioperative chemotherapy in locally advanced gastric cancer (complete and subtotal regression rate of 37% vs. 23% *p* = 0.02) [31]. In the setting of gastric cancer with CY+, there is to our knowledge a lack of prospective randomized data about the superiority of one chemotherapeutic regimen compared to another so far. In our series, we observed a conversion rate from CY+ to CY− with FLOT of 82% (9/11) and no patient converted under ECF (0/3). Based on the trend towards a higher conversion rate in our series and on the available data about advanced gastric cancer, we conclude that patients with gastric cancer with CY+ should benefit from the FLOT regimen in the neoadjuvant setting. We are aware of our limited numbers of patients with CY+, which is itself a rare condition and we think that further multicentric studies with larger cohorts of patients should be performed to achieve stronger results.

Another interesting observation is the recurrence pattern, which obviously depends on the peritoneal cytology status. It is well known that advanced stage gastric cancer is an independent prognostic factor of peritoneal recurrence, which is reported in the current literature ranging from 8 to 62% [14, 32–34]. In contrast, early-stage gastric cancer shows more often hematogenous recurrence patterns [35], and similarly, patients with CY− seem to have less peritoneal recurrence [14, 34]. In our series, recurrence rates in patients without peritoneal disease are comparable with the current literature [14]. We would like to highlight that patients with CY+ at any time had a higher risk to develop peritoneal recurrence, which indicates the need for adjuvant or additive therapy. Even for the patients who did convert to CY−, the recurrence rates are higher compared to the negative group. Although the mechanisms governing the occurrence of peritoneal metastasis and peritoneal recurrence remain unclear, we can postulate that based on the “seed and soil theory” the higher rate of peritoneal recurrence in the converted group is likely due to tumor cells that seed in the peritoneum and migrate through the basement membrane to the subperitoneal space, surviving therefore after neoadjuvant treatment and remaining undetected during peritoneal lavage [20]. Indeed, there is a meta-analysis of 20 randomized controlled trials which demonstrates the benefit of an adjuvant intraperitoneal chemotherapy after radical surgery in advanced gastric cancer, although a separate analysis investigating the role of adjuvant intraperitoneal chemotherapy in patients with CY+ and no visible peritoneal metastasis was not performed [36]. Recently, a phase II trial using a more aggressive approach combining CRS, gastrectomy, and HIPEC demonstrated encouraging results in patients with minimal peritoneal metastasis from gastric cancer or positive lavage cytology [37]. Some authors recommend even prophylactic HIPEC at the time of resection for advanced stage gastric cancer without peritoneal disease, highlighting the importance of reducing the risk of occult peritoneal dissemination during surgery in order to prevent future recurrence [38]. Currently, we may speculate that additive locoregional treatment in patients with CY+ might be considered in all patients undergoing resection, irrespective of their conversion status.

Another important issue that should be considered is the predicting factors that meant to achieve the best outcome possible. Our findings suggest that conversion of CY+ to CY− could be a prognostic factor for a better outcome. In the current literature, we can find some other important predictors that should be considered in the therapeutic decision-making. For example, peritoneal carcinomatosis itself appears to be a major driver of complications and dismal outcome for surgical gastrojejunostomy and should call for caution in the surgical strategy [39]. Moreover, an interesting study by Chen including 518 patients highlights the importance of the body mass index as another predicting factor for patients who have gastric cancer with peritoneal dissemination, especially in those who received palliative chemotherapy [40]. Other studies showed for example that lymphopenia and thrombocytosis were predictive factors for peritoneal seeding [41, 42]. Furthermore, recent studies try to identify new predicting factors such as the systemic immune-inflammation index aiming for a better oncological outcome and quality of life of our patients [43].

We would like to acknowledge the limitations of this study, which is a monocentric retrospective study. Furthermore, our analysis is based on a small number of patients with CY+ and without macroscopic peritoneal disease.

In conclusion, the overall survival of patients who convert to CY− after neoadjuvant chemotherapy may be superior compared to non-converted patients. Surgical treatment in well-selected patients should therefore be considered. However, peritoneal recurrence remains frequent despite conversion, urging for a better local control.

## Data Availability

The datasets used and analyzed during the current study are available from the corresponding author on reasonable request.

## Abbreviations

CY+: Positive peritoneal cytology
CY−: Negative peritoneal cytology
FLOT: 5-Fluorouracil plus leucovorin, oxaliplatin, and docetaxel
ECF/X: Epirubicin, cisplatin, and 5-fluorouracil/capecitabine
CRS: Radical cytoreductive surgery
HIPEC: Hyperthermic intraperitoneal chemotherapy
PIPAC: Pressurized intraperitoneal aerosol chemotherapy
OS: Overall survival
DFS: Disease-free survival
